# Adverse Reactions to Foods and Food Allergy: Development and Reproducibility of a Questionnaire for Clinical Diagnosis

**DOI:** 10.1155/2013/920679

**Published:** 2013-10-01

**Authors:** Nilza R. S. Lyra, Maria E. F. A. Motta, Luiz A. R. Rocha, Dirceu Solé, Décio M. Peixoto, José A. Rizzo, Luis Taborda-Barata, Emanuel S. C. Sarinho

**Affiliations:** ^1^Universidade Federal de Pernambuco (UFPE), Centro de Pesquisas em Alergia e Imunologia Clínica (HC/UFPE) Av, Professor Moraes Rêgo s/n, Cidade Universitária, 50670-420 Recife, PE, Brazil; ^2^Universidade Federal de São Paulo Departamento de Pediatria, Disciplina de Alergia Imunologia Clínica e Reumatologia Rua dos Otonis 725, V Clementino, 04025-002 São Paulo, SP, Brazil; ^3^Universidade da Beira Interior, Centro de Investigação em Ciências da Saúde, Avenida Infante Dom Henrique, 6200-506 Covilhã, Portugal

## Abstract

*Objective*. To develop a questionnaire as a screening tool for adverse reactions to foods in children and to assess the technical reproducibility by test-retest. *Methods*. Reproducibility of the questionnaire was performed by the literature review, preparing the preliminary questionnaire, peer review, pretest, and retest analysis. The study of the test-retest reproducibility was cross-sectional and descriptive. Kappa coefficient was used to study the reproducibility of the questionnaire. The sample consisted of 125 2–4 year-old children from 15 daycare centers in Recife, Brazil, and interviews with parents or caregivers were used to collect data. *Results*. From the total children, sixty-three were boys (50.4%), forty-six were two years old (36.8%), forty-seven were three years old (37.6%), and thirty-two were four years old (25.6%). Forty caregivers reported that their child had health problems with food. Most frequently reported offending foods were milk, peanuts, shrimp, and chocolate. Nine questions showed a good Kappa index (≥0,6). *Conclusions*. The questionnaire used needs to be resized and reshaped on the basis of the issues with good internal consistency and reproducibility. The use of a validated and reproducible questionnaire in the children represents an important contribution towards assessing an eventual rise in overt food allergy.

## 1. Introduction

An adverse food reaction consists of any abnormal reaction to the ingestion of food or additives, and it can be either toxic or nontoxic. Non-toxic adverse food reactions are related to the individual susceptibility and include food allergy, in which there is participation of immunological mechanisms which may or may not involve immunoglobulin E (IgE) [1, 2].

There has been an increase in the incidence of food allergy and a change in patterns of its presentation in several countries, especially in the most developed ones. For some authors the hygiene theory may be involved, which postulates that environmental changes in the industrialized world would induce a lower frequency of contact with infectious agents at an early age, thereby resulting in an increase in the tendency towards developing allergic diseases [3, 4].

In any case, rates of food allergy vary according to the studied population, its age, customs, or local diet, among other factors [5]. The prevalence observed in children under three years of age is estimated, on the basis of various studies, to range between 5% and 8%, and from 1% to 2% in adults [6–10]. In an epidemiological survey carried out in Brazil, suspected food allergy was detected in 7.3% of the patients seen by pediatric gastroenterologists [11]. 

In contrast with what happens with other allergic diseases such as asthma and rhinitis, for which there are many consistent studies regarding their natural history, prevalence, symptoms, and trends, food allergy has thus far been poorly studied regarding these aspects [12, 13]. 

The diagnosis of food allergy is made from detailed clinical history and physical examination, together with the analysis of the response to an exclusion diet of the suspect food and, in selected cases, to the oral challenge test, which is regarded as the gold standard for diagnosis. Laboratory and imaging tests are required depending on the signs and symptoms, and they further complement the diagnostic investigation [14–16]. 

Thus far, several studies analysing the prevalence of food allergy have been using questionnaires as an initial approach, followed by skin prick tests, determination of food-specific IgE levels, and/or oral provocation test for diagnosis [17–20]. 

The construction of a reproducible questionnaire using the correct methodological steps may be a low cost instrument to be applied to a greater section of a population as an initial approach for the identification of individuals with suspected food allergies. Furthermore, the validation of such an instrument should underlie the full diagnostic process through which individuals with suspected food allergies may be subsequently evaluated by a gold standard—the double-blind, placebo-controlled food challenge tests. 

The criteria for a study to be considered as scientific are reliability and validity. Reliability (reproducibility, reliability, repeatability, or precision) consists of matching results from the same research tool used in quantitative studies, applied by different researchers and/or at different times, namely, when the same measurement is repeated [21]. However, the criterium of validity (accuracy or precision) refers to the ability of the instrument to measure the true value of what is proposed or, in other words, if the results represent the truth or how much it moves away from it [22]. 

The psychometric property most commonly used to measure reproducibility is the test-retest method, which consists of applying the research tool to the same respondents at different times, under identical conditions and application methods [21, 23]. Although the test-retest reproducibility aims at the measurement of stability and can measure the variation due to the instrument [21], it may also introduce variations of the individual especially when the time interval between measurements is wide [23]. In any case, the reproducibility or reliability of the applicators is demonstrated when two researchers use the same instrument on the same individuals and achieve the same results, thereby obtaining a higher reliability of the instrument [21].

In order to elaborate a questionnaire, some steps should be completed, ranging from the theoretical-methodological basis, planning, and development of the questions to the statistical treatment of the data, aiming at the construction of a research tool that can be useful for analysis of a population. 

In Brazil, there is the need of a well-designed, reproducible, and validated questionnaire that can be used for the initial diagnosis of adverse food reactions and putative food allergies. Therefore, the objective of this study was to construct and test the reproducibility of a questionnaire for identification of individuals with suspected adverse reactions to foods and food allergy, with a view to its postvalidation use in the community as an initial approach to the studying of food allergies. 

## 2. Methods

The first stage of this study was composed of a bibliographical survey about the existence of validated questionnaires for research on food allergy in childhood, based on Internet databases connected to MEDLINE, such as Bireme, SciELO, and PubMed. Some descriptors were used, such as survey, and food allergy, questionnaire, food hypersensitivity. However, since none of the questionnaires used in the detected literature reports were described, there are, as far as we know, no available reports of standardized questionnaires to be used for the study of food allergies in children. 

Although some questionnaires in foreign languages were found and could be cross-culturally adapted, they might not always be applicable to other populations, because of social, ethnic, and religious differences, thereby compromising the quality of the data obtained [24]. It was then decided to construct the questionnaire used in this study.

The draft was based upon the main clinical manifestations of the adverse reactions to foods and food allergy. The variables were thus obtained from clinical history data of these diseases, which remains the basis of the diagnosis. The following aspects were contemplated: identification of suspected food and how much of it was ingested; time elapsed between ingestion and the onset of symptoms; whether the suspect food intake caused similar symptoms at other times or not; if there were other concurrent factors, such as physical exercise; and when the last reaction occurred. 

The design of the questions was objective, in order to obtain the most accurate reported evidence. The introductory questions were simple and motivating in order to raise the interest and willingness of the respondents to participate in the interview. The other questions were based on previous publications about the topic and were related to common manifestations of food allergy, aiming at defining features that might guide their diagnosis within the group of adverse reactions to foods. 

This initial version was submitted for review by a committee of six pediatrician experts who were postgraduated in the areas of allergy and gastroenterology, with clinical experience in food allergy. The committee could suggest modifications deemed relevant and also suggest the inclusion of aspects not covered by the questionnaire. Furthermore, this panel also analysed the semantics as well. 

After making the changes suggested by the expert panel, the next step was pretesting the questionnaire by conducting an exploratory pilot study which consisted of the application of questionnaires on 20 patients with previously confirmed food allergies and who attended a specialized clinic at the Hospital of the Federal University of Pernambuco. This step aimed at improving the instrument by analysing how laypeople with food allergy who deal with the problem and may have some knowledge about this type of allergy understand the questions of the instrument. In the meantime, while the pilot study was being applied, some modifications were also made to the survey. Together, these analyses allowed the construction of the final questionnaire whose questions were tested for reproducibility (Table 1). 

The calculation of the sample for the cross-sectional study was carried out using the EPI INFO 6.0 software and taking into consideration the literature data which showed a prevalence of about 6% of food allergy in children in the first 3 years of life. With the value of 1.4% as the acceptable minimum value and a confidence interval of 95%, the sample should consist of 102 children. Facing the possibility of sample loss or difficulties in accessing respondents, 125 2–4 year old children were randomly recruited from daycare centers in Recife. 

Interviews were conducted at established schedules, according to the availability of their parents or guardians. There was full cooperation of the children's families and of the staff of the units, especially when retests took place on weekends. 

Before proceeding with the interview, the researchers were introduced to the parents or caregivers and explained the objectives of the study and the duration of the interview. In case the parents or guardians allowed the children to participate in the study, a written consent form was given, read, and signed in duplicate by the parents or guardians and the researcher. 

In order to calculate the reproducibility of the test, it was established that 25% of the sample would be submitted to a retest 48 hours after the interview and that the children who were randomly selected among the ones who were initially selected for the interview (test) were scheduled to the retest. Thus, 35 children were considered for the retest. 

For the test-retest reproducibility analysis the Kappa concordance test and its respective confidence intervals (95%) were applied. The Kappa values are considered good when they were equal to or greater than 0.60 [22, 25]. 

The research protocol of this study was approved by the Research Ethics Committee involving human beings of the Center for Health Sciences of the Federal University of Pernambuco, in accordance with Resolution 196/96 of the National Health Council, under Protocol no. 035/2005. 

## 3. Results 

The study population comprised 125 children of a low socioeconomic status and with a balanced sex ratio: 63 were boys (50.4%), and 62 were girls (49.6%). Of the children, 46 were two years old (36.8%); 47 three years old (37.6%), and 32 four years old (25.6%). 

Table 1 shows the reproducibility of several questions about the adverse food reaction together with the associated Kappa index. The analysis of the consistency of the reactions to food ingestion was made in accordance with the highest frequency of citation. In 50% of the cases, the Kappa values obtained were substantial or perfect while in the remainder values were slight, fair or moderate. 

Among the 125 individuals responsible for the children (parents or guardians), 40 (32.0%) responded affirmatively to question “A”: “Do you think your child has a health problem with some food (or drink)?” Six of two or more incriminated food as described in Table 2. The Kappa index for questions “A” and “B” were substantial (0.64 and 0.70, resp.).

The questions with the highest levels of agreement which presented good reproducibility and which should remain to be tested in the Validation Study are shown in Table 3. 

**Figure 1:**
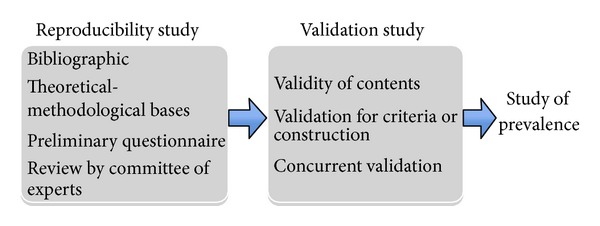
Flowchart of reproducibility and validation studies leading to subsequent epidemiological studies of adverse reactions to foods and food allergy.

Figure 1 contains the necessary steps already partly covered and those still to occur to validate the survey.

## 4. Discussion

In the present study, we constructed and tested the reproducibility of a questionnaire for identification of individuals with suspected adverse reactions to foods and eventual food allergy, for the first time in the Portuguese language, in order to subsequently validate it for use in community studies of food allergies. Our preliminary study allowed us to discriminate between questions with good or low reproducibility, thereby allowing us to subsequently delete and/or modify some of the questions. Finally, our preliminary study allowed us to detect a relatively high number of individuals (32%) who reported adverse food reactions.

Some study limitations can be attributed to certain aspects observed during the preparation and application of the questionnaire, such as the large number of questions and the use of open questions or items with open answers which made the application take longer, especially when the respondent claimed adverse reactions to more than one food. The large number of questions with the objective of contemplating all the aspects of food allergy did not increase the reliability of the data collected, and, in fact, it was reflected in the low concordance of some of the questions. Despite having been clearly and understandably written for the majority of the interviewees, some questions were poorly reproducible or even discordant and should not have been used. This limitation could be assigned to the source of information bias and memory of past events, since most of the respondents demonstrated total cooperation with the interview. 

Regarding the adverse food reaction and foods most frequently cited in this study, 32% (40/125) of the interviewees claimed to have had a health problem upon ingestion of some food, which is in accordance with the data of the literature. In fact, an earlier study showed that approximately 25% of the population of the United States believes that it has allergic food reactions [26]. Furthermore, according to a European study, self-reported intolerance to any food, including adverse reactions such as hives or other allergic symptoms after eating a particular type of food, have been described in up to 35% of the studied children [27]. In any case, currently it is estimated that the real prevalence of food allergy in Western populations is much lower. For instance, in the American population, food allergy is confirmed by history, and oral provocation tests range between 2 and 8% for infants and less than 2% for adults [26]. In fact, it is known that the prevalence of self-reported food allergy in studies in which questionnaires are used is much higher as compared to that found using objective measures of diagnosis [28]. The different estimates found between patients' self-perception and the real prevalence are attributed to the greater number of cases of food intolerance, mistakenly regarded as food allergy [12]. 

In terms of foods implicated in self-reported adverse food reactions, milk was the most frequently incriminated food in our study, with 30% (12/40) of the interviewees claiming it was the suspect food, probably because it is one of the most consumed at the age group studied. Peanut was the second, mentioned by 22.5% (9/40) of the interviewees. Although it is known that the consumption of peanuts varies according to regional habits of each population, an increase in its worldwide prevalence has been observed, with higher rates in Westernized countries, especially in the United States, United Kingdom, Canada, and Australia [29]. Shrimp, which was mentioned by 20% (8/40) of the respondents, is part of the diet of the low socioeconomic status population, even in the age group of our study. It is recognized as a highly perishable food, and therefore it may also be responsible for nonimmunologically mediated adverse reactions. 

Our preliminary study showed that the reproducibility of some of its questions was not high. In this regard, questions “C” and “D” are part of the group of questions about adverse reaction. The question regarding the initial exposure to the offending food (item “C”) showed a regular Kappa index in the questionnaire retest, and it should not be used because it induces the memory bias of the respondents, with the possible exception of individuals who suffer from IgE-mediated allergies in well-defined intense situations. On the other hand, question “D,” about how much time after eating the food did the child present reactions (either less than two hours or after two hours), showed regular Kappa indexes (0.44 and 0.49) in both analyzed aspects. In this context, the information to be obtained about the length of time between the ingestion and the clinical picture, although relevant, may not be that easily remembered by the respondents. 

The questions about ingestion of the same food by the other household members and the occurrence of symptoms (questions “E1” and “E2”) showed poor Kappa indexes (0.25 and 0.27, resp.) and were not useful since the children spend most of their time in a daycare center where they have most (five) of their meals each day. 

The question about the clinical picture induced by the offending food (question “F”) showed a good Kappa index (0.71). The answers were grouped for purposes of analysis of concordance, according to the list and spontaneous quotes on respiratory, cutaneous, and gastrointestinal tract manifestations. Manifestations reported upon ingestion of the suspected food may contribute towards the diagnosis of adverse food reactions and eventually also enhance the suspicion index of possible IgE-mediated food allergies. 

Question “G” was useful for the suspected cases of IgE-mediated reactions, with perfect concordance (*k* = 100), thereby simulating a cutaneous test where the food itself works as an extract. 

In terms of questions related to the treatment of the reported food-induced reactions, it is clear that visits to the hospital are easily remembered (question “H,” which showed a good Kappa index). Curiously, as far as information regarding the administration/use of medication, although the Kappa index was regular in a hospital environment (question “I”) possibly since most parents were unaware of what had been administered at health care centers, it was good when the medicine was given at home (question “J”). 

Questions about reexposure to the offending food and the recurrence of the clinical reactions (questions “L” and “M”) showed regular and good Kappa indexes, respectively, Question “O”, regarding the appearance of new symptoms upon re-exposure had a negative Kappa index, which is in agreement with absence of concordance. 

The open question about when was the last time that the reaction occurred (item “O”) showed a low Kappa index (0.28). This may be due to the fact that most reactions which occurred were not severe which makes it less likely for a child or a parent to remember the date or time when the event took place. 

The question regarding food restriction (item “P”) showed a good Kappa index (0.63). In fact, apart from being common practice among mothers to avoid some food they believe to “do harm” to their children, it is also a general consensus, among laypeople, that an exclusion diet is the chosen treatment for all food allergies [10]. 

Question “Q”, about the occurrence of pruritus, swelling, or tingling of the mouth when in contact with fruit or raw vegetable, showed perfect concordance with a Kappa index of 1.00. 

Although the application of questionnaires for the diagnosis of food allergies might not fully reflect their actual epidemiology, it is an important screening tool for such diagnosis, apart from eventually being a guide for planning health-related actions of health services in general and specialized medicine [12]. 

In Brazil, there are no epidemiological data about the prevalence of adverse food reaction and food allergy in children since there are no population-based studies that have used questionnaires which have been analyzed in terms of reproducibility and validation. 

In our study, we were able to confirm that the questionnaire we developed showed a good degree of reproducibility. Nevertheless, it is clear that the questionnaire needs to be resized and reshaped. The questions with good internal consistency and good levels of reproducibility highlight the major aspects of an adverse food reaction with a possibly allergic etiology. However, this study also suggests that a less extensive questionnaire, reformulated with questions with higher levels of concordance should be produced and subsequently subjected to further validation. Thus, we propose that the questions shown in Table 3 (A, B, F, G, H, L, M, P, and Q) should be further validated in children. 

As described in Figure 1, our questionnaire will therefore need to be further validated, and its content should be further analysed to confirm that it is appropriately designed in to reach its objectives, namely, in terms of its construct, and to check whether there is correlation between the included questions and also whether it is in concordance with available knowledge. Besides these steps, the questionnaire will subsequently have to be validated in terms of sensitivity and specificity indexes by comparison with the reference gold standard for diagnosis of food allergy, which is the double-blind, placebo-controlled oral provocation test [21, 22]. 

The use of a reproducible and validated questionnaire in a pediatric population will represent an important contribution to the approach to a problem that is on the rise in the Western world, which is food allergy.

## Figures and Tables

**Table 1:** Evaluation of the test-retest reproducibility of the survey about adverse food reaction and food allergy according to Kappa concordance.

Variable	Observed concordance		Kappa value (95% CI)
	*N*	%	
Age	35 (35)	100.0	1.00 (1.00-1.00)
Gender	35 (35)	100.0	1.00 (1.00-1.00)
*Adverse food reactions *			
(A) Do you think your child has any health problems with any food?	28 (35)	80.0	0.64 (0.37–0.90)
(B) Which food do you think your child has any reaction to?	30 (35)	85.7	0.70 (0.47–0.94)
(C) Did the reaction take place the first time you gave your child this type of food?	28 (35)	80.0	0.55 (0.29–0.80)
(D1) How long after eating did it take for the reaction to occur (up to 2 hours)?	27 (35)	77.1	0.44 (0.14–0.73)
(D2) How long after eating did it take for the reaction to occur (more than 2 hours)?	27 (35)	77.1	0.49 (0.20–0.77)
(E1) Did anybody else eat the same food?	28 (35)	80.0	0.25 (−0.16–0.68)
(E2) Did someone else also have the same reaction upon food ingestion?	31 (35)	88.6	0.28 (−0.25–0.80)
(F) What was the reaction your child had after eating this food?	30 (35)	85.7	0.71 (0.47–0.94)
(G) Has your child had any reaction when this food only touched her skin?	35 (35)	100.0	1.00 (1.00-1.00)
(H) Was there any need to seek medical care in a hospital?	31 (35)	88.6	0.67 (0.40–0.93)
(I) Did your child have to be given any medicine in the hospital?	29 (35)	82.8	0.55 (0.24–0.87)
(J) Did your child have to take any medicine at home?	28 (35)	80.0	0.58 (0.28–0.88)
(L) After the reaction did your child eat the same food again?	28 (35)	80.0	0.65 (0.40–0.90)
(M) Did your child have the same reaction when she ate the same food again?	32 (35)	91.4	0.76 (0.55–0.98)
(N) Did your child have another reaction upon eating the same food again?	31 (35)	88.6	−0.08 (−0.35–0.19)
(O) When was the last time your child had a reaction to this food?	24 (35)	68.6	0.28 (−0.04–0.62)
(P) Did your child stop eating the food after having a reaction?	28 (35)	80.0	0.60 (0.33–0.87)
(Q) Did your child feel itching, swelling, or numbness in his/her mouth after eating any fruit or raw vegetable?	35 (35)	100.0	1.00 (1.00-1.00)

*The questions listed above were written in a language which made them understandable for the family members.

**Table 2:** Distribution of frequency of responses of the 125 respondents about the occurrence or not of adverse food reaction (question “A”) and identification of foods reported by 40 respondents as being associated with symptoms (question “B”).

Variable	*N*	%
(A) Do you think your child may have a health problem (reaction) related to food (or drink)?		
Yes	40	32.0
No	85	68.0
Total		
(B) Which food or drink do you think your child has any reaction to?		
Milk	12	9.6
Peanut	9	7.2
Shrimp	8	6.4
Chocolate	5	4.0
Other	10	2.8
Frequency of adverse food reaction	44	65.2
Total	40	32.0

**Table 3:** Questions with higher index of concordance proposed for the evaluation of validity.

Questions	Kappa value	Interpretation of Kappa value
(A) Do you think your child may have a health problem (reaction) related to food (or drink)?	0.64	Substantial agreement
(B) Which food or drink do you think your child has any reaction to?	0.70	Substantial agreement
(F) What reaction did the child have after eating this particular food (in order of higher frequency of each food)?	0.71	Substantial agreement
(G) Has your child had any reaction when this food only touched her skin?	1.00	Perfect agreement
(H) Was there any need to seek medical care in a hospital?	0.67	Substantial agreement
(L) Did your child eat the same food again?	0.65	Substantial agreement
(M) Did your child have the same reaction upon eating the same food again?	0.76	Substantial agreement
(P) Did your child stop eating the suspect food?	0.60	Moderate agreement
(Q) Has your child had any reaction upon eating any fruit or raw vegetable?	1.00	Perfect agreement
